# Epidemiology and factors associated with peripheral neuropathy among HIV infected patients in Gondar, Ethiopia: A cross-sectional study

**DOI:** 10.1371/journal.pone.0211354

**Published:** 2019-01-29

**Authors:** Kedir Sany Adem, Balamurugan Janakiraman, Berihu Fisseha Gebremeskel, Mulugeta Bayisa Chala, Asmare Yitayeh Gelaw, Kassahun Alemu

**Affiliations:** 1 Department of Physiotherapy, College of Medicine and Health Sciences, University of Gondar, Gondar, Ethiopia; 2 Department of Physiotherapy, School of Medicine, College of Health Sciences and Ayder comprehensive specialized Hospital, Mekelle University, Mekelle, North Ethiopia; 3 Department of Epidemiology and Preventive Medicine, Monash University, Melbourne, Victoria, Australia; 4 Department of Epidemiology and Biostatistics, Institute of Public Health, College of Medicine and Health Sciences, University of Gondar, Gondar, Ethiopia; The Ohio State University, UNITED STATES

## Abstract

**Background:**

Antiretroviral therapy has surely increased the life expectancy of people living with HIV. However, long term complications like HIV associated sensory neuropathy has a negative impact on quality of life among people living with HIV (PLHIV). In Ethiopia, lack of data on magnitude of the burden and predictors of HIV associated sensory neuropathy in many resource limited setting has led to under diagnosis and eventually under management of HIV-SN. Hence, this study was set out to establish the burden of HIV-associated sensory neuropathy and, its association with demographic, health and clinical characteristics among people living with HIV in Ethiopia.

**Methods:**

Cross-sectional study was conducted to assess the prevalence of HIV-associated sensory neuropathy and the associated factors among adult HIV patients at University of Gondar Teaching Hospital, Gondar, Ethiopia. Brief Peripheral Neuropathy Screening tool validated by AIDs Clinical trial group was used for screening HIV-associated sensory neuropathy. Data were analyzed descriptively and through uni- and multivariate logistic regression.

**Results:**

In total 359 adult PLHIV with a mean age of 36.5± 9.07 years participated, their median duration of HIV infection was 60 months (IQR 36–84) and their median CD4 count 143cells/μL (IQR 69.5–201.5). Age above 40 years, anti-tuberculosis regimen, tallness, and exposure to didanosine contained antiretroviral therapy were found to be associated with HIV-associated sensory neuropathy (AOR 1.82, 1.84, 1.98 and 4.33 respectively).

**Conclusions:**

More than half of the HIV patients who attended HIV care clinic at University of Gondar hospital during the study period were found to present with peripheral sensory neuropathy. Higher age, tallness, TB medication, and didanosine in ART were significantly associated with HIV-SN as screened by effective diagnostic (BPNS) tool.

## Background

Life expectancy of people living with HIV has considerably increased after antiretroviral therapy (ART) era making HIV infection a chronic illness [1]. An estimated 5.5 HIV infected people among 6.6 million those who were saved from death between 1995 and 2012 live in low-middle income countries [2], [3]. Many debilitating complications related to HIV infection and ART regime are the recent spotlight [4]. HIV-associated sensory neuropathy (HIV-SN) is one of those complications among people living with HIV and the most common cause of chronic neuropathic pain, loss sensation, paraesthesia, foot ulcers, unemployment, poor, patient adherence to treatment, follow-ups, and poor quality of life [5–8]. The absence of neuro-regenerative therapies and proven ineffectiveness of analgesics in treatment of neuropathic pain among patients with HIV-SN clearly demonstrates the lack of effective treatments and the need for early diagnosis of patients at risk of HIV-SN [9–12].

Approximately 50% of the HIV- infected individuals receiving ART in developing countries reside in sub-Saharan [2], [3], [13]. Although there is an on-going decline in HIV-associated CNS disease and opportunistic infections in recent years, peripheral sensory neuropathies associated with HIV and ART remains to be high and frequent [14], [15].

The global estimate of HIV-SN varies widely from 1.73 to 69.4% in different HIV infected population. Studies done in resource-limited settings in sub-Saharan Africa, reported the risk of HIV-SN in ART patients with a range of estimates, from 30% to as high as 64% and the constraints in treatment availability in these settings [1], [5], [16]–[18]. These differences probably reflect the use of varying definitions of peripheral neuropathy and lack of use of a validated screening tool to diagnose HIV-SN [19], [20]. Hence, this study used Brief Peripheral Neuropathy Screening (BPNS) tool validated by AIDs Clinical trial group[21].

Moreover, the development of HIV-SN is likely to be influenced by various demographic, bio-clinical characteristics, among patients with HIV and some first-line drugs of ART. In Ethiopia, where the prevalence of adult HIV has been estimated to be 1.1% or nearly 1.2 million PLHIV [22], the burden of HIV-SN has not yet been estimated. Furthermore, the ART regime contains drugs some of which have been demonstrated in other studies to increase the risk of HIV-SN [23]–[26]. Therefore, this study was set to determine the burden of HIV-SN of the lower extremity, and the associated demographic, health and clinical characteristics, among PLHIV attending HIV care clinic at University of Gondar hospital (UOGH), North West Ethiopia.

## Methods

### Study design, setting, and participants

An institutional based cross-sectional descriptive study was carried out from February 2017 to June 2017. UOGH HIV care clinic is located in Gondar town, Ethiopia. UOGH is a 700 bedded governmental referral hospital that serves poor both urban and rural community. The HIV care and treatment started in 2003. As of 2016, the clinic cares for 11,127 HIV infected patients that is about 90 to 130 patients every day. This institution provides HIV testing including CD4 cell count monitoring every 6 months, health care consultation, counseling, and ART medication for those with CD4 count ≤ 350 cells/mm^3^ free of cost. Ethical approval was obtained from the Gondar University School of Medicine research and ethical review committee (SOM-2310706). Consent form was explained and written consent was obtained from all the participants.

Patients of both sexes attending UOGH HIV care clinic and aged ≥18 years were eligible for inclusion. All the participants signed written informed consent agreeing on participation and permitting to use their medical records/files. From the information, a total of 29 participants with additional medical conditions like; active tuberculosis (TB) patients and receiving treatment during study period (37.9%), cognitive disorders (3.4%), history of peripheral nerve injury (10.3%), chronic renal conditions (13.8%), active opportunistic infection (17.2%), leprosy (3.4), vitamin B12 deficiency (10.3), severe communication impairments (3.4), and were excluded from this study. Among the 29 excluded participants, 9 diabetic HIV patients with additional medical conditions like current TB, and renal disease were excluded.

### Sample determination and sampling procedure

The sample size required for this study was determined using single population proportion formula by assuming the prevalence to be 35% from previous research [27], with a 95% confidence interval, and 5% marginal error. The derived sample size was n = 326. Accounting for an estimated non-response or refusal rate of 10%, the required sample size was n = 359.

**Fig 1:**
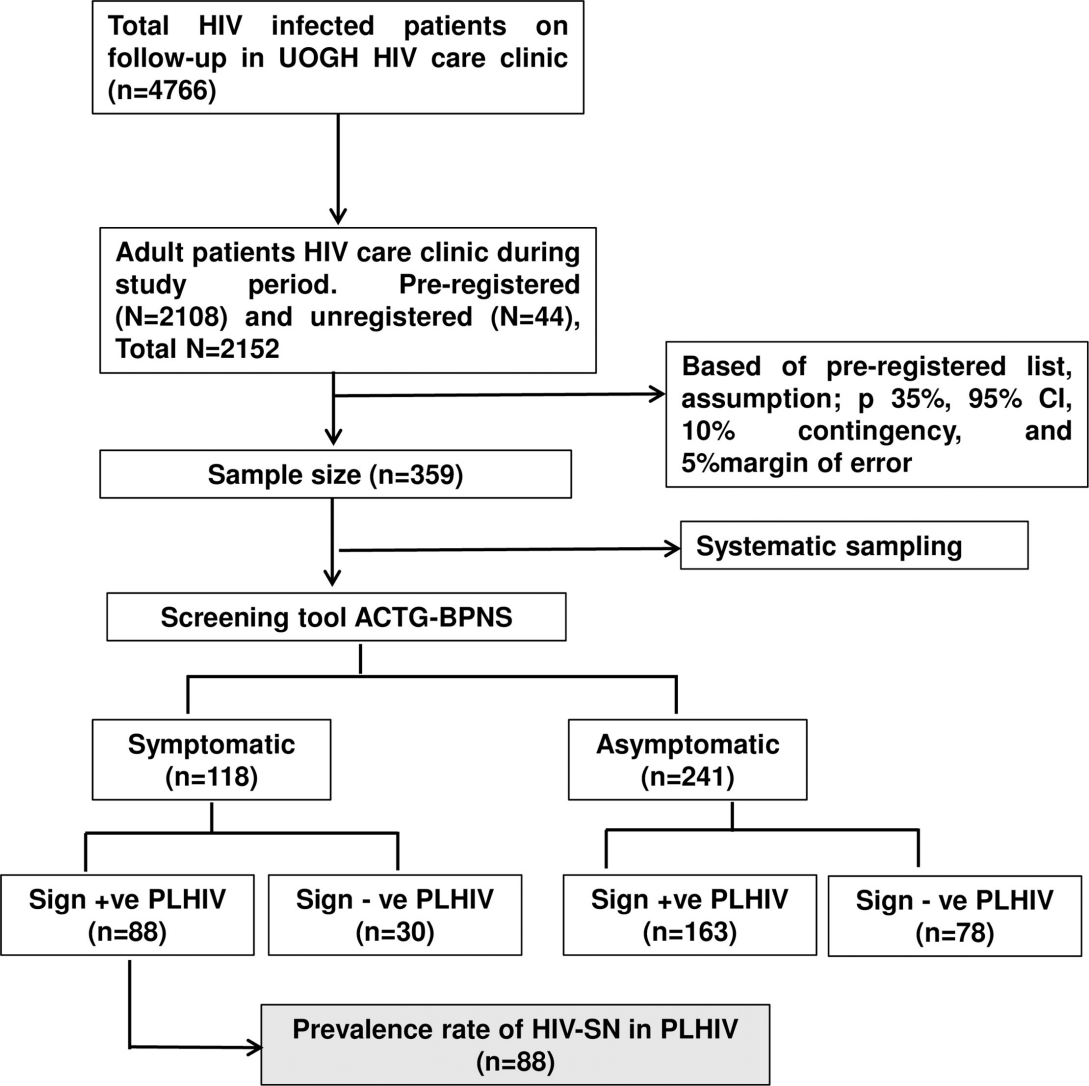
Study flow diagram. UOGH-University of Gondar Hospital, BPNS-Brief Peripheral Neuropathy Screening, PLHIV-People living with HIV, HIV-SN-HIV associated sensory neuropathy.

The study population was considered homogeneous and as indicated by the unpublished prior feasibility pilot study, a systematic random sampling was implemented. From the HIV-infected patients attending HIV care clinic each day during the study period, K^th^ patient was selected and the first person between 1 and K was randomly chosen, then taking every K^th^ number thereafter, where K^th^ was a sampling interval on the register list made for the day. The K^th^ (K = 6) was determined by dividing the total number of participants on the list (N), by the sample size. The procedure was repeated until estimated eligible sample size was reached; a sample of 359 was included from the total pre-registered number of 2108 for the month (Fig 1).

### Study procedure

#### Demographic and clinical data

Participants demographic, clinical and laboratory data were extracted from the medical chart and by using a purposely prepared structured questionnaire by trained nurses. Behavioural characteristics like alcohol consumption, smoking, khat-chewing, and physical activity were self-reported. Clinical staging based on WHO guidelines [28] were done as a part routine by clinicians. Physical measurements such as height and weight were measured and results of the screening were recorded onto a standardized form.

#### HIV-associated sensory neuropathy data

Prior to data collection, a two-day intensive training was given to data collectors (physiotherapist) by the principal investigator (KS) and clinical neurologist of UOGH following which a pre-test of data collection was carried out with 15 PLHIV. Brief peripheral neuropathy screening tool validated by the AIDS Clinical trial Group (ACTG) [20] was administered by a trained physiotherapist. In this screening, first patients were questioned to rate presence and severity of symptoms in feet and/or legs, using a scale of 1 (mild) to 10 (severe) for both legs separately. Symptoms included pain, aching, or burning; a sensation of ‘‘pins and needles” and numbness. The highest of the 3 scores were converted into a subjective PN grade, as follows: symptoms absent, grade 0; the score of 1–3, grade 1; the score of 4–6, grade 2; and score of 7–10, grade 3. Objective findings included in the BPNS were a loss of vibration perception and abnormal ankle deep tendon reflexes. Vibration perception was evaluated using a 128-Hz tuning fork, maximally struck and applied at the great toe distal interphalangeal joint of each foot.

Vibration sense was defined and graded as normal or grade 0 for vibration felt for >10seconds, as mild loss or grade 1 for vibration felt for 6–10 seconds, as moderate loss or grade 2 for vibration felt for < 5 seconds, and as severe loss or grade 3 for no feeling of vibration. Ankle reflexes were tested using a reflex hammer and graded as follow (0: absent, 1: hypoactive, 2: normal, 3: hyperactive, 4: clonus). Based on PNS validation study, the presence of HIV-SN was defined when someone had both subjective neuropathy (grade, >0) and ≥1 abnormal finding bilaterally on physical examination. A neurologist randomly examined (3 patients/day/30 days on random visits) the previously evaluated patients by the trained physiotherapist using BPNS to ensure accuracy and all were concordant.

### Data management and analysis

The collected data was re-checked for its completeness and entered into EPI info version 7.0 (Centres for Disease Control and prevention, USA) and then transferred to IBM Statistical Package for Social Sciences (SPSS) version 20.0 for Windows for statistical analysis. Descriptive statistics were examined to determine the representativeness of study sample to the research setting population during the study period in percentages, frequency, and margin of error. Formulas proposed by Cochrane [29] were used to calculate the normal approximation frequency [S1 File]. The magnitude and severity in relation to socio-demographic and health variables were analysed using descriptive analysis. HIV-SN was defined by the presence of symptoms and at least an abnormal perception of vibration or abnormal ankle reflexes or both and expressed as percentage of the study population.

With HIV-SN as the dependent variable (categorized; present or absent), bivariate and multivariate binary logistic regression analyses were executed to establish the association with different independent variables. Chi-square or appropriate Fisher’s exact test was used to analyse prevalence distribution of HIV-SN and estimate its association with categorized independent variables. Independent variables included in the model were age, gender, height, weight, level of education, marital status, occupation, place of residence, duration of HIV, current and baseline CD4 count, ART regimen combination, duration on ART, and previous/history of TB regimen. Model fit was also assessed by described method [30]. Results were considered statistically significant when 95% confidence intervals not containing unity (equal to *p-*value <0.05). Initially, bivariate analyses were conducted and independent variables that were found statistically significant were included in multivariate analysis. Crude and adjusted Odds Ratios (OR) together with its 95% Confidence Intervals (95%CI) was reported. Potential confounding variables were entered into the model as covariates and variability in association checked. When clear subgroups seemed present in the data, significance testing (Pearson χ^2^) and, if appropriately sized subgroups per category remained, logistic regression were performed.

## Results

### Patient characteristics

A total of 359 adult PLHIV consented and participated in the study and this is more than 100% of the power calculated sample size (n = 326). Of the 359 participants majority of them 234 (65.2%) were females. The mean age, weight and height were 36.5 + 9.07 years, 55 + 10.4kg, 1.61 + 0.82m respectively. In addition to those who had registered (N = 2108), forty four unregistered HIV patients attended HIV care clinic for consultations during study period. The average age of the population (n = 2152) was 48 years. Nearly one fourth (26.5%) of them were underweight, almost two in five (40.4%) were married, almost a quarter (25.6%) of them were uneducated, 68(18.9%) were governmental employees, 314 (94.7%) were Orthodox Christian and the majority (94.7%) of them live in urban area. None of the patient had diabetes. Most of the subject (93.6%) self-reported no previous or current alcohol consumption and almost all (98.6%) subjects reported no history of smoking Table 1. Except Pre-HAART participants 3.6%, the frequency of the remaining demographic and clinical variables of the research sample fell within the total population range (95% CI) and the research sample was determined to be representative of the research setting population (S1 File).

**Table 1:** Distribution of HIV-SN and socio-demographic characteristics of 359 patients living with HIV [PLHIV] who attended HIV care clinic, North West Ethiopia, 2017.

Variable		Sample total	HIV-SN	
			Present	Absent
		n (%)	n (%)	n (%)
All participants		359 (100%)	88 (24.5%)	271 (75.5%)
**Sex**	Male	125 (34.8)	29 (8.1)	96 (26.7)
	Female	234 (65.2)	59 (16.4)	175 (48.8)
**Age group (years)**	< 30	74 (20.6)	13 (3.6)	61 (17)
	30–39	177 (49.3)	38 (10.6)	139 (38.7)
	40–49	68 (18.9)	18 (5.01)	50 (13.9)
	50–59	32 (8.9)	14 (3.9)	18 (5.01)
	≥60	8 (2.2)	5 (1.4)	3 (0.8)
**Residence**	Urban	340 (94.5)	82 (22.8)	258 (71.7)
	Rural	19 (5.29)	6 (1.7)	13 (3.59)
**Marital status**	Married	145 (40.4)	37 (10.3)	108 (30.1)
	Single	45 (12.5)	7 (1.9)	38 (10.6)
	Divorced	85 (23.7)	15 (4.2)	70 (19.5)
	Windowed	71 (19.8)	25 (7.0)	46 (12.8)
	Separated	13 (3.6)	4 (1.1)	9 (2.5)
**Level of Education**	Uneducated	92 (25.6)	30 (8.4)	62 (17.2)
	Grade 1–6	70 (19.5)	14 (3.9)	56 (15.6)
	Grade 7–8	43 (12)	6 (1.7)	37 (10.3)
	Grade 9–10	59 (16.4)	16 (4.5)	43 (11.9)
	Grade 11–12	43 (12)	12 (3.3)	31 (8.7)
	College & above	52 (14.5)	10 (2.9)	42 (11.6)
**Occupational status**	Governmental	68 (18.9)	18 (5.0)	50 (13.9)
	Private employee	53 (14.8)	9 (2.5)	44 (12.3)
	Daily labourer	48 (13.5)	15 (4.2)	33 (9.3)
	House wife	66 (18.4)	14 (3.9)	52 (14.6)
	Unemployed	49 (13.6)	12 (3.3)	37 (10.3)
	Farmer	17 (4.7)	4 (1.1)	13 (3.6)
	Other	59 (16.4)	16 (4.5)	43 (11.9)
**Religion**	Orthodox	314 (87.5)	79 (22.0)	235 (65.5)
	Muslim	37 (10.4)	6 (1.7)	31 (8.7)
	Protestant	8 (2.2)	2 (0.5)	6 (1.7)
	Others	1 (0.3)	0 (0.0)	1 (0.3)
**Alcoholic history**	Yes	23 (6.4)	4 (1.1)	19 (5.3)
	No	336 (93.6)	84 (23.4)	252 (70.2)

The median duration of HIV infection since diagnosed as on 31st March 2015 was 60 months (IQR; 36–84).The use of highly active antiretroviral therapy (HAART) was also 60 months (IQR; 28–84). The median baseline and recent CD4 cell count were 143 cell/mm^3^ (IQR; 69.5–201.5) and 368 cell/mm3 (IQR; 246–546.8) respectively. 346 (96.4%) were on HAART, 158 (44%) were on a regimen containing AZT+3TC+NVP and majority of the subject 326 (90.8%) were in the clinical stage between I and II. Of the 359 patients, 160 (44.6%) participants reported a history of anti-TB treatment and 157 (43.7%) reported being on both anti TB and ART regimen in the past Table 2.

**Table 2:** Distribution of HIV-SN and clinical characteristics of 359 patients living with HIV [PLHIV] for adults aged 18 and above years, North West Ethiopia, 2017.

Variable	Sample total	HIV-SN	
		Present	Absent
	N (%)	n (%)	n (%)
**All participants**	**359 (100%)**	**88 (24.5%)**	**271 (75.5%)**
**Baseline CD4 cell count**			
> 200 cell/mm^3^	94 (26.2)	30 (8.36)	58 (16.16)
≤ 200 cell/mm^3^	263 (73.3)	58 (16.16)	205 (57.10)
**Recent CD4 cell count**			
≥ 350 cell/mm^3^	195 (54.3)	49 (13.65)	146 (40.67)
200–350 cell/mm^3^	100 (27.9)	21 (5.85)	79 (22.01)
≤ 200 cell/mm^3^	61 (17)	17 (4.74)	44 (12.26)
**WHO disease stages**			
Stage I and II	326 (90.8)	80 (22.28)	246 (68.52)
Stage III and IV	33 (9.19)	8 (2.23)	25 (6.96)
**History of anti TB treatment**			
Yes	160 (44.6)	51 (14.21)	109 (30.36)
No	199 (55.4)	37 (10.31)	162 (45.13)
**Type of HAART regimen**			
AZT+3TC+NVP	158 (44)	35 (9.75)	123 (34.26)
AZT+3TC+EFV	12 (3.3)	5 (1.39)	7 (1.95)
TDF+ 3TC+EFV	107 (29.8)	23 (6.41)	84 (23.4)
TDF+ 3TC+NVP	43 (12)	13 (3.62)	30 (8.36)
ABC+DDI+LPV/R	15 (4.2)	10 (2.79)	5 (1.39)
Others regimen	11 (3.1)	1 (0.28)	10 (2.795)
Pre-HAART	13 (3.6)	1 (0.28)	12 (3.34)

### HIV-associated sensory neuropathy

Using BPNS tool, the prevalence of neuropathic symptoms was found to be 32.9% (118/359) and the most common were the sensation of aching and burning type of pain on leg/ feet (Table 3). Among patients who self-reported symptoms, 32.2% had both diminished ankle reflexes and impaired perceptions of tuning fork vibration, 28.8% had only diminished ankle reflex, 13.5% (16/118) had only abnormal perception of vibration, 24.6% (30/118) had neither abnormal perception of vibration nor diminished reflex.

**Table 3:** Prevalence of sign and symptoms consistent with HIV-SN among adult HIV patient 2017, Ethiopia.

Symptoms (at legs or feet)	n (%)	
Pain, aching or burning	95(26.5)	
Pins and needles	24(6.7)	
Numbness (lack of sensation)	49(13.6)	
Asymptomatic	241(67.1)	
**Ankle reflex**	**Right leg**	**Left leg**
Grade 0: absent reflex	28(7.8)	28 (7.8)
Grade 1: hypoactive	169 (47.1)	167(46.5)
Grade 2: normal	152 (42.3)	153(42.6)
Grade 3: hyperactive	10 (2.8)	11(3.1)
Grade4: clonus	0(0)	0(0)
**Perception of tuning fork vibration**	**Right leg Left leg**	
Grade 0: max. perception > 10 sec	216 (60.2)	118 (52.4)
Grade 1: perception for 6–10 sec	113 (31.5)	145 (40.4)
Grade 2: perception for ≤ 5 sec	22 (6.1)	19 (5.3)
Grade 3: no perception	8 (2.2)	6 (1.7)

Among those without symptoms, 163 (68%) at the least reported either the impaired perception of vibration or diminished ankle reflex, 49 (20.3%) had both diminished ankle reflex and abnormal vibration perception, 76 (31.5%) had only diminished reflex, 38 (15.7%) had only abnormal vibration perception. The prevalence of HIV-SN according to ACTG-BPNS definition among the PLHIV was 24.5% (88/359) of the total study samples Table 1. The median duration of symptoms reported was 12 months (IQR; 2–42). Among asymptomatic, 21% of participants were found to be normal using BPNS examination.

### Patient characteristics predicting prevalence of HIV-SN

HIV-SN in people living with HIV was significantly associated with patient characteristics such as advanced age, height, history of anti-tuberculosis treatment and didanosine (DDI) contain in HAART regimen. Thus, in this study HIV-infected patients with advanced age (> 40 years old) were nearly two times more likely to develop HIV-SN than younger (< 40 years old) patients [AOR = 1.82, 95% CI: 1.05,3.13], patients with history of anti- tuberculosis regimen were nearly two twice more likely to develop HIV-SN than no history of antituberculosis treatment [AOR = 1.84, 95% CI: 1.09,3.10], patients with increased height(> 170 cm) were very nearly two folds more likely to develop HIV-SN than reduced height (< 170 cm) [AOR = 1.98, 95% CI: 1.01,3.91], and DDI contain in HAART regimen were more than four times more likely to develop HIV-SN in HIV-infected patients [AOR = 4.33, 95% CI: 1.34,14.00] Table 4. Hence, age, height, history of TB medication, and DDI in HAART were found to be major predictors of HIV-SN in this study.

**Table 4:** Univariate and multivariate analysis for the associated and predicting demographic and health status characteristics to HIV-SN, 2017, Ethiopia.

Variables	HIV-SN		Univariate	p	Multivariate	p
	Yes	No	COR (95% CI)		AOR (95%,CI)	
**Age**						
≤40 years	51	200	1(ref)		1(ref)	
>40 years	37	71	2.04(1.23,3.37)	0.005	**1.82(1.05,3.13)**	0.031*
**Height**						
≤170 cm	68	240	1(ref)		1(ref)	
>170 cm	20	31	2.27(1.22,4.24)	0.01	**1.98(1.01,3.91)**	0.047*
**History of anti TB treatment**						
No	37	162	1(ref)		1(ref)	
Yes	51	109	2.04(1.25,3.33)	0.004	**1.84(1.09,3.10)**	0.021*
**Duration with HIV since diagnosis.**						
≤5 years	38	146	1(ref)		1(ref)	
>5years	50	125	1.53(0.94,2.49)	0.082	**1.24(0.43,3.5)**	0.68
**Duration of ART**						
≤5 years	43	152	1(ref)		1(ref)	
>5years	44	107	1.45(0.89,2.36)	0.133	1.01(0.35,2.9)	0.974
**DDI contain in HAART regimen.**						
No	77	254	1(ref)		1(ref)	
Yes	10	5	6.59(2.18,19.88)	0.001	**4.33(1.34,14.00)**	0.014*

*Denotes significant association of characteristics with HIV-SN in multivariate model, AOR- Adjusted odds ratio, CI -Confidence Interval, COR-Crude odds ratio

## Discussion

The BPNS as an screening tool in examining HIV-SN in HIV-infected patients has been validated using physiologic (quantitative sensory threshold testing) and pathologic (epidermal nerve fiber density) testing as the gold standards [20]. Definition of HIV-SN consistent with BPNS tool is presence of both subjective and objective findings. However, the criteria of BPNS that a patient must have both self-reported neuropathic symptoms and clinical signs to be classified as HIV-SN may eventually result in omission of some patients with mild HIV-SN [31]. With this diagnostic criterion of BPNS tool, there are always chances for substantial underestimation of peripheral neuropathy (PN) [32].

Using BPNS screening and definition of HIV-SN used in the validation study, the prevalence of HIV-associated sensory neuropathy in our study participants was 24.5%. A study which used clinical criteria (signs and symptoms of neuropathy) along with more standardized electrophysiology studies reported that two-thirds of HIV-SN is subclinical [33]. This means even if an asymptomatic patient present with abnormal clinical signs relevant to HIV-SN may not fulfill the diagnostic criteria for HIV-SN as per the BPNS tool. Similarly, in our study 45.7% (163/359) of asymptomatic patients had an abnormal perception of clinical signs relevant to HIV-SN. Some authors [4], [33], [34] categorize this group as asymptomatic PN or early HIV-SN and also suggest that they are more likely to become symptomatic when exposed to risk factors.

However, the most striking finding in this study is, if we are to combine both the group of patients identified with HIV-SN as per ACTG-BPSN definition and asymptomatic PN with relevant clinical sign, the prevalence of PN in our study will alarmingly rise to 70.2%. In addition, a further 10% PLHIV reported neuropathic pain with absences of objective findings indicating early HIV-SN and they are more likely to become symptomatic when challenged with other risks for HIV-SN. Which suggest that the prevalence will expand to near 80% and only a mere 20% patients seems to be unaffected with normal examination using BPNS tool. This method of identifying PN will surely reduce the chances of underestimation by BPNS tool and will improve chances of early detection of PN among HIV in the low resource setups like UOGH.

A study done among rural dwellers in Rwanda [15] reported a prevalence of 40% peripheral neuropathy from one rural district hospital. But, the Rwandan study used the Subjective Peripheral Neuropathy Screen (SPNS) that evaluates HIV-SN subjectively, unlike BPSN that evaluates signs objectively in addition to the subjective assessment. Furthermore, the prevalence found in this study is lower than studies done in Africa, such as Jimma, Ethiopia 34.6%, Nigeria 39% and Mombasa, Kenya 36% [18], [25], [27], [35]. A similar prevalence was found in studies done in Douala, Cameron 21% [34] and in Tanzania 20.9% [36]. The possible reasons might be due to differences in methodologies, characteristics of study population, exposure to risk factors, sample size, intervening variables and unidentifiable environmental factors. Moreover, the prevalence of HIV-SN reported in this study falls lower than the range of 30 to 60% reported elsewhere. Even so, it is important to note that as much as 85% of HIV-infected patients in Ethiopia are on ART and aging (2) and some ARTs regimen along with other coexisting-risk factors are likely to influence the development of HIV-SN. Eventually, the prevalence of HIV-SN in Ethiopia is more likely to continue increasing. In contrast, factors like less ART-naïve patients, better managed HIV, and frequent screening for HIV-SN may result in decline of HIV-SN rate. Thus efforts are needed to frequently identify the prevalence of HIV-SN so as to develop cost-effective strategies to curb the burden.

Similar to other studies [15], [32], [37] our study identified higher age (> 40 years) to be a predictor variable for developing HIV-SN. With the rapidly aging HIV population and improved life expectancy due to successful therapy may further increase the burden of HIV-SN and amplify the challenges in HIV therapeutics.

Our study confirms the clinical notion that HIV-SN in HIV infected patients is height dependent, with a small, but the consistently significant HIV-SN risk in taller HIV-infected patients. The height of > 170 cm was two times more likely to develop HIV-SN when compared with patients <170 cm [AOR = 2.144, 95% CI: 1.076, 4.269], which is consistent with other sub-Saharan studies [16], [34], [36], [38]. Unlike other studies [17], this study demonstrated no association between gender (men), weight and HIV-SN. Gender association deserves concern in future studies done in Ethiopia.

Low enrolment of PLHIV with a known history of diabetes, history of alcohol consumption and smoking had limited the power to evaluate their association and possible interaction effect of diabetes mellitus with HIV infection in our study where several other studies have suggested a strong association. Moreover, in resource-limited settings, diabetes is often not diagnosed early and the possibility of undiagnosed diabetes among PLHIV prevails. History of anti TB medication and concurrent use of ART was found to be strongly associated to HIV-SN in our study with isoniazid being the most common anti TB drug administered. Though isoniazid exposure is reported as risk factor elsewhere [34], its sole association with HIV-SN is beyond the scope of the data set of this study.

In contrast to many studies [23], [24], [27], this study found out that, HIV-SN with or without symptom did not associate with low CD4 counts or greater WHO stage of the disease. Limited enrolment of patients with low CD4 counts and the criteria to commence ART had been revised to CD4 count < 350 cells/mm^3^might be reasons behind it. Previous studies [25], [26] also reported that after successful therapy neither CD4 count nor viral load suppression decreased the odds of developing peripheral neuropathy. Several studies [10], [17], [39] postulate D-drugs related mitochondrial toxicity as a cause of HIV-SN. The majority of our study population were on HAART for more than 6 years and previous exposure to stavudine may also have contributed.

A few limitations can be mentioned to benefit future research and the findings must be considered in the light of these limitations; this study includes its definition of SN which was chosen based on previous work validating the ACTG-BPNS but resulted in patients who had isolated neuropathic symptoms or asymptomatic signs being classified as neuropathy free. We were not able to distinguish DSP and ATN due to the limited enrolment of the pre-ART patient and a longitudinal design, unlike our cross-sectional design would enable us to determine temporal relationships. Furthermore, the non-availability of golden standard electrophysiological devices limited diagnostic accuracy. Despite these limitations, this study included a relatively larger representative sample of the clinical population, Ethiopia, which enhances external validity of results and provides a well-powered insight and estimate of prevalence of HIV-SN in resource limited settings.

## Conclusions

HIV-SN was found to be highly prevalent among HIV infected patient at the University of Gondar Hospital, Ethiopia As the life expectancy of people with HIV increases the prevalence of HIV-SN will also increase, making it a major clinical issue and burden particularly in resource limited settings. It is known that managing neuropathic pain is a challenging problem in HIV infected. Hence, early detection of HIV-SN permits patient education, control of modifiable risk and confounding factors, and better chances of improvement with added choices of managements. Simple and validated neurological assessment tools to detect early HIV-SN or those at risk among all HIV patients should be used routinely. We found that the occurrence of HIV-SN increases with advanced age and height thereby necessitating early diagnosis in these risk groups. Physician’s drug choice in HAART and the anti-tuberculosis regime shall consider the role of these drugs and possible association with HIV-SN especially in Sub-Saharan terrains.

### Ethical approval

Ethical approval was secured from the Ethical Review Committee of the College of Medicine and Health Sciences, University of Gondar, Ethiopia.

## Supporting information

S1 File Characteristics of study population and study sample. S1 File Legend Sample representativeness.
(PDF)

## Abbreviations

ACTG: AIDs Clinical trial group
AOR: Adjusted odds ratio
ART: antiretroviral therapy
ATN: antiretroviral therapy toxic neuropathy
BPNS: Brief Peripheral Neuropathy Screening
cART: combination antiretroviral therapy
CI: Confidence Interval
DDI: didanosine
HIV-SN: HIV associated sensory neuropathy
HIV-DSP: HIV associated distal sensory polyneuropathy
HAART: highly active antiretroviral therapy
IQR: Inter-Quartile Range
PLHIV: People living with HIV
PN: Peripheral neuropathy
SN: Sensory neuropathy
TB: Tuberculosis
