# Supplementary material for: Epidemiology and factors associated with peripheral neuropathy among HIV infected patients in Gondar, Ethiopia: A cross-sectional study
Source: PLoS One. 2019 Jan 29;14(1):e0211354. doi: 10.1371/journal.pone.0211354 (PMC6350981; doi:10.1371/journal.pone.0211354)
Supplement: S1 File — Legend Sample representativeness. (PDF) [file pone.0211354.s001.pdf]

Supporting file: Sample representativeness

Table 1 Socio-demographic characteristics of the population (N 2152) of the research setting and sample (n 359) patients living with HIV [PLHIV] for adults aged 18 and above years, North West Ethiopia, 2017

Variable		Sample total	Population		E(np)	Population 95% CI
		n (%)	N	%		
**All participants**		**359 (100%)**	**2152**	**100%**	**N**	
**Sex**	Male	125 (34.8)	782	36.3	130.5	34.2, 38.5
	Female	234 (65.2)	1,370	63.7	228.5	63.0, 67.4
**Age in years, mean**		36.5	36.84			35.4, 37.5
**Age group (years)**	< 30	74 (20.6)	396	18.4	66.1	16.7, 21.0
	30-39	177 (49.3)	1105	51.3	184.3	50.4, 54.7
	40-49	68 (18.9)	390	18.1	65.1	16.4, 20.7
	50-59	32 (8.9)	221	10.3	36.9	8.3, 12.7
	≥60	8 (2.2)	40	1.9	6.7	-0.3, 4.1
**Residence**	Urban	340 (94.5)	1959	91.0	326.8	91.0, 95.4
	Rural	19 (5.29)	193	9.0	32.2	7.0, 11.4
**Marital status**	Married	145 (40.4)	828	38.5	138.1	37.2, 41.6
	Single	45 (12.5)	261	12.1	43.5	10.2, 14.6
	Divorced	85 (23.7)	522	24.3	87.1	22.7, 27.0
	Windowed	71 (19.8)	471	21.9	78.6	20.2, 24.6
	Separated	13 (3.6)	70	3.3	11.7	1.2, 5.5
**Level of Education**	Uneducated	92 (25.6)	569	26.4	94.9	24.9, 29.2
	Grade 1-6	70 (19.5)	451	21.0	75.2	19.3, 23.6
	Grade 7-8	43 (12)	247	11.5	41.2	9.6, 13.9
	Grade 9-10	59 (16.4)	340	15.8	56.7	14.0, 18.4
	Grade 11-12	43 (12)	277	12.9	46.2	11.0, 15.4
	College & above	52 (14.5)	268	12.5	44.7	10.6, 14.9
**Occupational status**	Governmental	68 (18.9)	410	19.1	68.4	17.3, 21.7
	Private employee	53 (14.8)	298	13.8	49.7	12.0, 16.4
	Daily labourer	48 (13.5)	304	14.1	50.7	12.3, 16.6
	House wife	66 (18.4)	382	17.8	63.7	16.0, 20.4

	Unemployed	49 (13.6)	328	15.2	54.7	13.4, 17.8
	Farmer	17 (4.7)	89	4.1	14.8	2.1, 6.4
	Other	59 (16.4)	341	15.8	56.9	14.0, 18.4
**Religion**	Orthodox	314 (87.5)	1908	88.7	318.3	88.6, 93.0
	Muslim	37 (10.4)	175	8.1	29.2	6.1, 10.5
	Protestant	8 (2.2)	46	2.1	7.7	0.1, 4.4
	Others	1 (0.3)	23	1.1	3.8	-1.1, 3.3
**Alcoholic history**	Yes	23 (6.4)	149	6.9	24.9	4.9, 9.3
	No	336 (93.6)	2003	93.1	334.1	93.1, 9.5

Formula proposed by Cochrane E(np): Unbiased estimate of normal sample representation.
Cochran, W.G. (1977). *Sampling techniques.* (3rd ed.). NewYork, NY: Wiley.

Table 2 Clinical characteristics of the population (N 2152) of the research setting and sample (n 359) patients living with HIV [PLHIV] for adults aged 18 and above years, North West Ethiopia, 2017

Baseline CD4 cell count	Sample total n (%)	Population N (%)	E(np)	Population N 95% CI
> 200 cell/mm^3	96 (26.7)	632 (29.4)	105.4	27.2, 31.6
$\leq$ 200 cell/mm^3	263 (73.3)	1520 (70.6)	253.6	68.4, 72.8
**Recent CD4 cell count**				
$\geq$ 350 cell/mm^3	195 (54.3)	1194 (55.5)	199.2	53.3, 57.7
200-350 cell/mm^3	100 (27.9)	567 (26.3)	94.6	24.1, 28.5
$\leq$ 200 cell/mm^3	61 (17)	391 (18.2)	65.2	16.0, 20.4
**WHO disease stages**				
Stage I and II	326 (90.8)	1974 (91.7)	329.3	89.5, 93.9
Stage III and IV	33 (9.19)	178 (8.3)	29.7	6.1, 10.5
**History of anti TB treatment**				
Yes	160 (44.6)	991 (46.1)	165.3	43.9, 48.3
No	199 (55.4)	1161 (53.9)	193.7	51.7, 56.1
**Type of HAART regimen**				
AZT+3TC+NVP	158 (44)	984 (45.7)	164.2	43.5, 47.9
AZT+3TC+EFV	12 (3.3)	64 (3.0)	10.7	0.8, 5.2
TDF+ 3TC+EFV	107 (29.8)	568 (26.4)	94.8	24.2, 28.6
TDF+ 3TC+NVP	43 (12)	209 (9.7)	34.9	7.5, 11.9
ABC+DDI+LPV/R	15 (4.2)	91 (4.2)	15.2	2.0, 6.4
Others regimen	11 (3.1)	90 (4.2)	15.0	2.1, 6.5
Pre-HAART*	13 (3.6)	146 (6.8)	24.4	**4.6, 9.0**

*sample % doesn't fall within 95% CI range of population

Example: Using unbiased estimate of normal sample representation by Cochrane (1) formula;

- **For gender: Male**
- Formula: V(np) _ nP(1-P)*(1 _ f)
- Where n = sample size = 359
- P = actual proportion = 782/2152
- nP = sample count = 125
- = multiplication
- 1_f _ % of observations not in the sample = [1793/(2102 - 1)]
- V(np) = 359*(782/2152)(1370/2152)*(1793/2151) = 68.6
- ***E(np) is an unbiased estimator of (757/2102)*359 = 130.5***
- ***Our male sample was n- 125 male which approximates 130***

Percentages of variable were nearly similar for both sample and population, And unbiased estimator showed close proximity as well, suggesting that the sample was representative of the clinical population.

Reference

1. Cochran WG. Sampling Techniques: 3d Ed. Wiley New York; 1977.

Using the Margin of Error method (95% CI)

To know whether our sample is representative of the population, Frequency and percentage of categorical variables were run. To figure out the margin of error, we divide 1 by the square root of (Population N) 2152 and multiply by 100.

Square root of 2152 = 46.39

1/Square root of 2152 = 0.02155

0.022*100 = **2.16%.** This means that there should be a 95% chance that our population parameter is within 2.16% of our statistics.

Example:

Gender: n 359

Male: [n = 125], [N 782]

36.3% - 2.16 = 34.2
36.5% + 2.16 = 38.5 [95% CI Range 34.2, 38.5]

Sample parameter of Male % is 34.8 which is within the range. So, male sample represent our clinical population.
